# Influence of the COVID-19 pandemic on the epidemiological profile of the initial care of victims of falls

**DOI:** 10.1590/0100-6991e-20233422-en

**Published:** 2023-02-17

**Authors:** LUIZ CARLOS VON-BAHTEN, ALIANA LUNARDI ZVICKER, ANGEL ADRIANY DA SILVA, BEATRIZ ZANUTTO SALVIATO, HELOÍSA MORO TEIXEIRA, PAULA KAORI ANDO, RAFAELLA STRADIOTTO BERNARDELLI

**Affiliations:** 1 - Hospital Universitário Cajuru, Liga Acadêmica do Trauma (LATHUC) - Curitiba - PR - Brasil; 2 - Universidade Federal do Paraná, Departamento de Clínica Cirúrgica - Curitiba - PR - Brasil; 3 - Pontifícia Universidade Católica do Paraná, Escola de Medicina e Ciências da Vida - Departamento de Clínica Cirúrgica - Curitiba - PR - Brasil; 4 - Pontifícia Universidade Católica do Paraná, Escola de Medicina e Ciências da Vida - Departamento de Bioestatística - Curitiba - PR - Brasil

**Keywords:** Epidemiology, Accidental Falls, COVID-19, Trauma Centers, Epidemiologia, Acidentes por Quedas, COVID-19, Centros de Traumatologia

## Abstract

**Objective:**

to assess the epidemiological profile of trauma patients from fall from the same level (FSL) and fall from an elevated level (FEL) during the COVID-19 pandemic, and to compare it with data from different levels of restriction (flags) and data prior to the pandemic.

**Method:**

a cross-sectional study with a probability sample of the medical records of patients aged 18 years or older admitted to the emergency room due to falls, from June 2020 to May 2021. Epidemiological data, such as sex, age and injuries were analyzed, as well the current level of restriction. The three restriction periods were compared between then and the proportion of admissions due to falls was compared with the period from December 2016 to February 2018.

**Results:**

a total of 296 admissions were evaluated, 69.9% were victims of FSL and 30.1% of FEL. The mean age was 57.6 years, and 45.6% were over 60 years old. Admissions among men predominated, and 40.2% of patients required hospitalization. During the red flag period, there were proportionally more injuries to the head and neck (p=0.016), injuries to extremities (p=0.015) and neurological trauma (p<0.001). An average of 6.1, 6.3 and 5.2 admissions per day was obtained during the yellow, orange and red flag, respectively. There was a relative increase in falls when compared to the pre-pandemic period.

**Conclusions:**

there was an absolute reduction in admissions of victims of falls in midst of the most restrictive period during the pandemic. However, when compared to pre-pandemic data, there was a relative increase in falls.

## RESUMO

**Objetivo:**

avaliar o perfil epidemiológico do trauma por quedas de mesmo nível (QMN) e quedas de nível elevado (QNE) durante a pandemia da COVID-19, realizar a comparação dos dados entre os níveis de restrição (bandeiras) e comparar com dados prévios à pandemia.

**Método:**

estudo transversal com amostragem probabilística de prontuários de pacientes com 18 anos ou mais admitidos na sala de emergência devido a quedas de junho de 2020 a maio de 2021. Foram avaliados dados epidemiológicos, como sexo, idade e lesões resultantes, além da bandeira vigente. Os três períodos de restrição foram comparados entre si e a proporção de atendimentos por quedas foi comparada com o período de dezembro de 2016 a fevereiro de 2018.

**Resultados:**

avaliou-se 296 atendimentos, sendo 69,9% vítimas de QMN e 30,1% de QNE. A média de idade foi 57,6 anos, sendo que 45,6% apresentavam idade superior a 60 anos. Sexo masculino predominou e 40,2% dos pacientes necessitaram internamento hospitalar. Durante a bandeira vermelha proporcionalmente ocorreram mais lesões em cabeça e pescoço (p=0,016), trauma em extremidades (p=0,015) e neurológico (p<0,001). Obteve-se uma média de 6,1, 6,3 e 5,2 atendimentos/dia durante a bandeira amarela, laranja e vermelha respectivamente. Ocorreu um aumento significativo da ocorrência de quedas quando comparado ao período prévio à pandemia.

**Conclusões:**

durante o período pandêmico, verificou-se uma redução absoluta de atendimentos de vítimas de quedas na bandeira mais restritiva. Porém, quando comparado ao período pré-pandêmico, verifica-se um aumento significativo das quedas.

## INTRODUCTION

A fall is an unintentional displacement of the body to the same level or a lower level than the initial position, caused by multifactorial circumstances, resulting in harm or not^1^. In the world, it leads to the death of around 640,000 individuals annually^1^, and in Brazil, between 1998 and 2015, it led to the hospitalization of 1,192,829 elderly people^2^.

Those most likely to suffer falls that require hospitalization are workers, athletes, and mainly the elderly, who account for 37.3 millions of serious falls^1^ ^,^ ^3^. They can cause both injuries (lacerations, fractures, and intracranial hemorrhages) and psychological damage (depression and anxiety). In addition, they have an impact on mortality, hospitalization rates, and medical care costs^4^.

Another disease generated similar impacts, the one caused by SARS-CoV-2, COVID-19. With the first case reported at the end of 2019, it has a broad clinical spectrum, from an asymptomatic condition to acute respiratory failure^5^. Due to its high transmissibility and the exponential increase in the number of infected people, it was considered by the World Health Organization (WHO) as a global pandemic in March 2020^5^. To contain the viral spread and avoid the collapse of health services, restriction measures were adopted in 190 countries^6^. The use of masks, hand hygiene, restriction of movement of people, and isolation of suspected and confirmed cases of COVID-19 were some of them^7^ ^,^ ^8^.

In this context, the Municipal Health Secretariat of Curitiba (State of Paraná, Brazil) established in June 2020 the Sanitary and Social Responsibility Protocol. This measure is composed of different levels of restriction (flags), implemented according to epidemiological indicators of COVID-19 advancement, incidence on the population, and the capacity of health services to provide care^9^. The yellow flag, signaling an alert, indicates milder restrictive measures. The orange flag, indicating moderate risk, applies restrictions on hours and capacity of services and events. The red flag, which indicates high risk, only allows essential services to operate and restricts the movement of people^9^.

During the pandemic, there was a reduction in attendances at trauma centers around the world associated with the implementation of similar measures^10^ ^-^ ^12^. With the reduction in the circulation of people, there was also a relative increase in accidents at home^10^ ^,^ ^11^, especially due to falls^10^ ^,^ ^12^ ^,^ ^13^ and in elderly populations^10^ ^,^ ^12^ ^,^ ^13^.

In view of the recurrence of this trauma mechanism and the importance of public health care in any context, including emergency situations, it is essential to constantly update the epidemiological data on this event. Therefore, the objective of this study is to outline the epidemiological profile of trauma due to falls in a hospital in Curitiba-PR during the pandemic, to evaluate its presentation in the different levels of restriction, as well as to compare it with pre-pandemic data from the same institution.

## METHODS

This is a cross-sectional study with probabilistic sampling of medical records of patients admitted to the emergency room of a university hospital in Curitiba-PR, from June 2020 to May 2021. We included patients aged 18 or older admitted to the emergency room by direct search or taken by medical rescue services. We excluded incomplete medical records and those of patients who died on arrival at the hospital.

The calculation of the sample size was based on the initial sample of 100 successive consultations that occurred during the pandemic and on data from a previous study carried out at the same institution in a period prior to the pandemic (December 2016 to February 2018)^14^, whose design, variables, and analysis are similar and whose results will be used as pre-pandemic data. In order to detect a significant difference between the distributions on the trauma mechanism classifications (falls, traffic accidents, and assaults), when comparing the pre-pandemic and during the pandemic periods, a total of 833 patients would be necessary, considering the significance level of 5% and test power of 80%. For the sampling of the medical records, we used the GraphPad software. Alternate days were selected within the study period and, on each day, one fifth of the medical records were drawn according to the inclusion criteria.

The variables collected from the medical records were age, sex, weekday or holiday, type of medical rescue service, fall mechanism, being fall from the same level (FSL) and fall from an elevated level (FEL), values resulting from the Glasgow Coma Scale (GCS), systolic blood pressure (SBP), and respiratory rate (RR) for the calculation of the Revised Trauma Score (RTS), patient comorbidities, work accident, self-reported description of alcohol consumption before the accident, extremity trauma, traumatic brain injury (TBI), suicide attempt, need of admission, and the period of the pandemic in which it occurred, considering the levels of restrictive measures implemented (flags). Curitiba has two main pre-hospital care services, the Integrated Emergency Trauma Care Service (SIATE) and the Mobile Emergency Care Service (SAMU). SIATE exclusively assists trauma victims together with the fire department^15^, while SAMU assists clinical emergencies and complements trauma care^14^. The RTS is a physiological score that assesses the morbidity and mortality of polytrauma victims. Its values vary between 0 and 8 and the higher the final value, the better the patient’s prognosis^16^.

Age results were described by mean, standard deviation, minimum and maximum, RTS by median, minimum and maximum value, and categorical variables by frequency and percentage. We used binary logistic regression models with statistical significance assessed by the Wald test to evaluate the explanatory potential of age for the occurrence of FSL, when compared with FEL, and to assess the association between the mechanism of trauma with the resulting injuries and the need for hospitalization. The models that showed statistical significance were expressed by Odds Ratio (OR) and its confidence interval. Using the Chi-square test, trauma victims the three groups established by the yellow, orange, and red flags regarding the mechanism of trauma (FSL or FEL), the presence of injury in one of the body segments, the need for hospitalization, and alcohol intake before the fall. For analyzes that showed statistical significance in the chi-square test, we performed multiple comparisons between groups, with the significance level corrected by Bonferroni. We also presented the Odds Ratio (OR) and its confidence interval for these two-by-two comparisons that were significantly different between groups. We used the Kruskal-Wallis non-parametric test to compare the RTS scores between groups according to the flags in force. We carried out two-by-two comparisons of the correlation between the period defined by the flags and the incidence of assistance to trauma involving a fall mechanism. We used the chi-square test to compare the proportions of consultations due to falls and others (traffic accidents and assaults) during the pandemic period with the proportions of consultations performed in the same institution during the pre-pandemic period^14^. For the analyzes that showed statistical significance in the chi-square test, we analyzed the residuals, considering that there was an association between the variables in the cells that have adjusted standardized residuals greater than 1.96. Values of p<0.05 indicated statistical significance. We organized the collected data in an Excel^®^ spreadsheet and analyzed them with the IBM SPSS Statistics v.20.0 software, IBM Corp, Armonk, NY. 

We adopted no strategies to correct missing data.

The research project was approved by the Ethics Committee under protocol CAAE n° 40014320.2.0000.0020.

## RESULTS

We included 296 patients. Of them, 69.9% had FSL, and 30.1%, FEL; 62.8% were male and the mean age was 57.6 years (± 20.4, range 18-98), 45.6% being over 60 years old (Table 1). We also observed that increasing age significantly augments the risk of sustaining FSL when compared with FEL (OR 1.042, 95% CI 1.028-1.057) (Table 2).

**Table 1:** Epidemiology of patients who sustained falls.

Variable	Classification	n=296 n(%)
Sex	Female	110 (37.2%)
	Male	186 (62.8%)
Age (years)	18-29	32 (10.8%)
	30-39	31 (10.5%)
	40-49	35 (11.8%)
	50-59	63 (21.3%)
	≥60	135 (45.6%)
Trauma Mechanism	Fall from the same level (FSL)	207 (69.9%)
	Fall from elevated level (FEL)	89 (30.1%)
Medical Rescue	SAMU	165 (55.7%)
	SIATE	116 (39.2%)
	Ambulance from the city of origin	6 (2.0%)
	Direct search	6 (2.0%)
	Helicopter	3 (1.0%)
Comorbidities	Systemic Arterial Hypertension	90 (30.4%)
	Diabetes mellitus	48 (16.2%)
	Cardiac insufficiency	5 (1.7%)
Variable	Classification	n=296 n(%)
	Epilepsy	10 (3.4%)
	Rheumatological diseases	6 (2.0%)
	Psychiatric disorders (anxiety, depression)	25 (8.4%)

**Table 2:** Trauma mechanisms according to age.

Mechanism	Victim’s age (years)					
	n	Average	Standard deviation	Minimum	Maximum	p*
FSL	207	62.3	19.8	18	98	<0,001
FEL	89	46.6	17.4	18	87	

FSL: Fall from the same level; FEL: Fall from elevated level. *Logistic Regression Model with significance assessed by the Wald test, p<0.05.

As for the medical rescue service, 55.7% of patients were brought to the hospital by SAMU and 39.2% by SIATE (Table 1). The days of the week in which there were more cases of falls were Thursdays, Fridays, and Saturdays, totaling 49% (n=145) of the visits.

Only 1.7% (n=5) of the cases were due to accidents at work. In addition, 20.3% (n=60) of the patients reported having consumed alcoholic beverages before the fall and 0.7% (n=2) tried to commit suicide with this event. Regarding comorbidities, systemic arterial hypertension was the most observed (30.4%), followed by type -2 diabetes (16.2%) (Table 1).

As for the affected body segment, 34.5% (n=102) of the patients had trauma to the extremities and 5.9% (n=6) of these had open fractures. In addition, 69 (23.3%) sustained TBI and 119 (40.2%) were admitted for surgery. The median RTS score of the total sample was 7.84, ranging from 5.97 to 7.84, with no significant difference scores between patients seen during the different flags (Yellow, Orange, or Red) of restriction during the pandemic (Table 3).

**Table 3:** RTS score in the different restriction levels (flags).

Flag	RTS score				
	n	median	Minimum value	Maximum value	p*
Yellow	90	7.84	5.97	7.84	
Orange	184	7.84	5.97	7.84	0.526
Red	22	7.84	6.90	7.84	

*Kruskal-Wallis non-parametric test, p<0.05.

Regarding the association between the trauma mechanism and the affected region, we found that FEL victims are more likely to suffer injuries to upper limbs (OR 2.53, 95%CI 1.39-4.63), to the chest (OR 4.82, 95% CI 2.04-11.39), to the back (OR 4.33, 95% CI 2.04-9.16), and to lower limbs (OR 1.70, 95% CI 1.01-2.87) when compared with FSL ones. There was no significant difference in the occurrence of specific injuries and the need for hospitalization between types of falls (Table 4).

**Table 4:** Affected body segment and need for hospitalization according to the type of trauma.

Variable	Trauma mechanism (group)			
	Presence	FSL (n=207) n (%)	FEL (n=89) n (%)	p-value*
Face injury	No	157 (75.8%)	66 (74.2%)	
	Yes	50 (24.2%)	23 (25.8%)	0.757
Head and neck injury	No	103 (49.8%)	49 (55.1%)	
	Yes	104 (50.2%)	40 (44.9%)	0.403
Upper limb injury	No	178 (86%)	63 (70.8%)	
	Yes	29 (14%)	26 (29.2%)	0.002
Chest injury	No	198 (95.7%)	73 (82%)	
	Yes	9 (4.3%)	16 (18%)	<0.001
Abdomen injury	No	206 (99.5%)	85 (95.5%)	
	Yes	1 (0.5%)	4 (4.5%)	-
Back injury	No	194 (93.7%)	69 (77.5%)	
	Yes	13 (6.3%)	20 (22.5%)	<0.001
Pelvis/hip injury	No	192 (92.8%)	81 (91%)	
	Yes	15 (7.2%)	8 (9%)	0.608
Lower limb injury	No	148 (71.5%)	53 (59.6%)	
	Yes	59 (28.5%)	36 (40.4%)	0.045
Injury to external surfaces	No	103 (49.8%)	49 (55.1%)	
	Yes	104 (50.2%)	40 (44.9%)	0.594
Injury to extremities	No	143 (69.1%)	51 (57.3%)	
	Yes	64 (30.9%)	38 (42.7%)	0.052
Open fracture	No	206 (99.5%)	84 (94.4%)	
	Yes	1 (0.5%)	5 (5.6%)	-
TBI	No	160 (77.3%)	67 (75.3%)	
	Yes	47 (22.7%)	22 (24.7%)	0.707
Need for hospitalization	No	127 (61.4%)	50 (56.2%)	
	Yes	80 (38.6%)	39 (43.8%)	0.406

FSL: Fall from the same level; FEL: Fall from elevated level. *Logistic Regression Model with significance assessed by the Wald test, p<0.05.

When comparing each of the variables with the occurrence at the different levels of restriction, we observed significant differences regarding the occurrence of head and neck injuries, injuries to external surfaces, trauma to the extremities, and TBI (Table 5). In the two-by-two comparisons, we found that those seen in the red flag had a significantly higher proportion of head and neck injuries (OR 3.529, 95% CI 1.307-9.529), extremity trauma (OR 3.972, 95% CI 1.506-10.477), and TBI (OR 13.333, 95% CI 4.484-39.649) compared with the yellow flag. In addition, the proportion of patients with TBI in the red flag was higher than in the orange one (OR 10.246, 95% CI 3.755-27.958) (Table 5).

**Table 5:** Variables studied at the different restriction levels.

Variable	Flag				
	Yellow (Y) (n=90) n (%)	Orange (O) (n=184) n (%)	Red (R) (n=22) n (%)	Overall p-value*	2-by-2 p-value #
Trauma Mechanism					
FSL	61 (67.8%)	130 (70.7%)	16 (72.7%)	0,850	
FEL	29 (32.2%)	54 (29.3%)	6 (27.3%)		
Facial injury	23 (25.6%)	48 (26.1%)	2 (9.1%)	0.211	
Head and neck injury	34 (37.8%)	95 (51.6%)	15 (68.2%)	0.016	Y vs. O: >0.05 Y vs. R: 0.030 O vs. R: >0.05
Injury to upper limbs	13 (14.4%)	35 (19%)	7 (31.8%)	0.166	
Chest injury	7 (7.8%)	17 (9.2%)	1 (4.5%)	0.728	
Abdominal injury	1 (1.1%)	4 (2.2%)	0 (0%)	-	
Injury to the back	6 (6.7%)	23 (12.5%)	4 (18.2%)	0.196	
Pelvic/hip injury	5 (5.6%)	16 (8.7%)	2 (9.1%)	0.641	
Injury to lower limbs	21 (23.3%)	65 (35.3%)	9 (40.9%)	0.089	
Injury to external surfaces	22 (24.4%)	55 (29.9%)	0 (0%)	0.010	Y vs. O: >0.05 Y vs. R:- ** O vs. R:- **
Trauma to extremities	24 (26.7%)	65 (35.3%)	13 (59.1%)	0.015	Y vs. O: >0.05 Y vs. R: 0.011 O vs. R: >0.05
Open fracture	1 (1.1%)	5 (2.7%)	0 (0%)	-	
TBI	15 (16.7%)	38 (20.7%)	16 (72.7%)	<0.001	Y vs. O: >0.05 Y vs. R: <0.001 O vs. R: <0.001
Need for hospitalization	32 (35.6%)	77 (41.8%)	10 (45.5%)	0.530	
Report of alcohol consumption	17 (18.9%)	40 (21.7%)	3 (13.6%)	0.622	

Result described in frequency (n) and percentage (%). *Chi-square test of the comparison between the three flags, p<0.05. #Comparison of groups two-by-two with significance value adjusted by Bonferroni correction. **Chi-square test not feasible, as there were cells with expected frequencies less than one.

As for the attendances according to the restriction level, there were on average 6.1, 6.3, and 5.2 visits during the yellow, orange, and red flags, respectively. There was a statistically significant difference when comparing orange and red flags (p=0.026), with a difference of 1.1 visits per day. There was no significant difference between the yellow and orange (p=0.628) and yellow and red (p=0.060) flags.

Regarding the comparison with the pre-pandemic period^14^, we observed a statistically significant difference in the proportions of trauma mechanisms between the data collection periods. When analyzing the standardized residuals, there was a significantly higher proportion of assistances to victims of falls during the pandemic (Table 6).

**Table 6:** Trauma mechanisms treated before ^*14*^ and during the COVID-19 pandemic.

Mechanism	Pre-pandemic (2016-2018)^14^	COVID-19 pandemic (2020-2021)	p-value*
	n (%) - [Residuals# ]	n (%) - [Residuals# ]	
Traffic accidents	658 (53.1%) - [2.49]	426 (47.7%) - [-2.49]	
Assaults	229 (18.5%) - [-0.44]	172 (19.2%) - [0.44]	0.031
Falls	352 (28.4%) - [-2.33]	296 (33.1%) - [2.33]	
Total	1,239 (100%)	894 (100%)	

*Chi-square test significance, p<0.05. # Adjusted standardized residuals: (observed frequency - expected frequency)^2^ ÷ expected frequency; cells with absolute values greater than 2 indicate significant association/difference between variables. Positive residuals indicate a direct relationship, while negative ones indicate an inverse relationship.

## DISCUSSION

When compared with the pre-pandemic period^14^, there was a significant increase in the proportion of visits due to falls. In addition, there was a predominance of FSL in relation to FEL. On the other hand, a French study found that, of the 151 cases of falls during the lockdown period, 84.8% were due to FEL and 15.2% to FSL^17^. Regarding age, most patients in this study were over 60 years of age and the greater the age, the greater the risk of FSL. A London study^12^ found a higher frequency of falls in the age group above 64 years (52.35%) and observed that this event was the most common trauma mechanism during the pandemic, representing 68.4% of cases^12^. Falls, especially FSL, were the main mechanisms of injury among the elderly in several studies^18^. The need for isolation of elderly patients in this context, due to the greater chance of developing serious conditions due to COVID-19, may have increased the risk of falls due to domestic accidents, which justifies the results of this study.

Among the main risk factors for falls are advanced age, physical disability, history of previous falls, and use of medication^19^. In addition to the prevalence of middle-aged patients in this study, we observed a high incidence of comorbidities, such as Hypertension and Diabetes Mellitus. A study^20^ with 706 elderly patients observed similar proportions, as well as other conditions such as heart disease (6.66%), rheumatological disease (3.56%), and epilepsy (2.17%)^20^. Hypertensive elderly people are approximately seven times more likely to suffer falls when compared with those who are not, as some antihypertensive drugs tend to cause postural hypotension. Diabetic elderly people, on the other hand, may present a decrease in sensorimotor function and neuromuscular and musculoskeletal deficits, which also increase the chance of falls^18^.

Most of the patients in this series were men, which differs from other series studies. In the pre-pandemic study of the same institution^14^, both in FSL and in car collisions, females predominated. Tiensoli et al.^18^ evaluated falls in elderly people aged over 60 years and also observed a predominance of females (66.92%). It is known that non-fatal falls are more common in women and the most common fractures occur in women who suffer this mechanism^19^. However, the occurrence of falls in men could be explained by the higher levels of risky and dangerous behaviors within occupations^21^, which corroborates the results found.

Alcohol intake prior to the accident occurred in 20.3% of the patients in this study, a value close to that found in other articles^22^. High levels of alcohol lead to a greater risk of falls due to imbalance and exposure to risk situations by reducing critical thinking^23^.

As for the medical rescue service, the proportion between the assistance provided by SAMU and SIATE in this study followed the pattern found in previous years^14^, showing the prevalence of the former. SAMU, in general, is more associated with clinical care, and SIATE is used by the population in accidents considered severe, such as traffic ones. In addition, some cases of falls may have been preceded by clinical situations, such as epilepsy or syncope, which could explain the greater activation of the SAMU. Also, the visits took place mainly on Thursdays, Fridays, and Saturdays, which also occurred in the pre-pandemic assessment (Fridays and Saturdays, 14.4% and 17.87%, respectively)^14^. It is important to be aware of the days on which most cases occur, as this can help prepare for medical care.

Regarding resulting injuries, trauma to the extremities occurred in less than half of the patients in this study. Moreover, injuries to the upper and lower limbs, thorax, and back were more likely in victims of FEL, a high-energy event, which is consistent with the literature^24^. A 2017 study^25^ reported that victims of falls had a higher frequency of pelvic fractures and spinal cord trauma than those involved in other events. Furthermore, 23.3% of patients had TBI in the present study. In the United States, contusions, hip fractures, or head trauma, which are considered moderate or severe injuries, are present in 20% to 30% of falls in the elderly. Furthermore, the greater the age, the greater the risks of severity of intracranial injuries^25^, and the absence of elderly-adapted environments can be an important factor for triggering accidents. Thus, the need for preventive measures for domestic accidents, especially for the elderly, has been highlighted, especially in the pandemic. Adequate lighting, care for uneven floors, ergonomic organization of furniture, and the use of non-slip shoes are some of them. As for medications, rational and spaced use is suggested, and the use of drugs for insomnia should be reduced or eliminated^10^.

When analyzing the restriction measures, we observed that the daily average of visits during the red flag decreased by 1.1 compared with the orange one. Other epidemiological studies have shown similar reductions in periods of greater restriction^26^ ^,^ ^27^.

One of the limitations of this study is that it was carried out in a single center, despite this being one of the reference hospitals in trauma care in the capital of Paraná. Thus, there is limitation regarding external generalization. In addition, the data referring to alcohol intake was the patients’ self-declaration, which may have resulted in underestimation due to omission.

## CONCLUSION

The epidemiological profile of victims of trauma due to falls during the COVID-19 pandemic was of elderly males falling from the same level. There was a reduction in the daily average of attendances under the most restrictive period and an increase in the proportion of cases of falls compared with the pre-pandemic period.
